# Psychosocial factors at work and common mental disorders in health care professionals and workers

**DOI:** 10.47626/1679-4435-2025-1421

**Published:** 2025-08-25

**Authors:** Mariana Aguiar do Vale, Evelyn Yamashita Biasi, Sergio Roberto de Lucca

**Affiliations:** 1 Psicologia, Centro Universitário de Adamantina, Adamantina, SP, Brazil; 2 Faculdade de Educação, Núcleo de Estudos do Trabalho, Saúde e Subjetividade (NETS), Unicamp, Campinas, SP, Brazil; 3 Faculdade de Ciências Médicas, Saúde Coletiva, Unicamp, Campinas, SP, Brazil

**Keywords:** health personnel, psychosocial impact, mental disorders, occupational health, pessoal de saúde, impacto psicossocial, transtornos mentais, saúde ocupacional

## Abstract

**Introduction:**

Psychosocial factors at work arise from the interactions between the organizational environment and the individual characteristics of workers, potentially affecting health, performance, and job satisfaction.

**Objectives:**

To investigate psychosocial work factors and the prevalence of psychological distress among health care professionals and workers in a general hospital.

**Methods:**

A convenience sample of 157 participants was analyzed. Quantitative data were collected using a sociodemographic questionnaire, the Health Safety Executive – Indicator Tool for assessing psychosocial work factors, and the Self-Reporting Questionnaire to screen for psychological distress. Qualitative data were gathered from three focus groups divided by job roles — nurses, nursing technicians, and administrative staff — totaling 20 volunteers.

**Results:**

The prevalence of common mental disorders among health care professionals and workers was 36%. An association was found between domains of the Health Safety Executive – Indicator Tool and psychological distress in health care professionals, particularly in the variables Colleague Support, Supervisor Support, and Communication. Among health care workers, this association was also observed in Demand, Relationships, Control, Supervisor Support, and Communication.

**Conclusions:**

Qualitative data highlighted the relevance of psychosocial factors in psychological distress. Sharing these findings with the health care organization is expected to lead to prompt concrete organizational and management actions to implement strategies to minimize psychosocial stressors affecting the health and well-being of workers.

## Abstract (pt)

**Introdução:**

Os fatores psicossociais no trabalho são elementos resultantes das interações entre o ambiente organizacional e as características individuais dos trabalhadores, podendo influenciar a saúde, o desempenho e a satisfação no trabalho.

**Objetivos:**

Investigar os fatores psicossociais no trabalho e a prevalência de sofrimento psíquico dos trabalhadores e profissionais de um hospital geral.

**Métodos:**

A amostra de conveniência foi de 157 participantes. Os dados quantitativos foram coletados a partir de um questionário sociodemográfico, o Health Safety Executive – Indicator Tool, que avalia os fatores psicossociais do trabalho, e o Self-Reporting Questionnaire, indicador de sofrimento psíquico. Os dados qualitativos foram obtidos a partir de três grupos focais, divididos por função: enfermeiros, técnicos de enfermagem e trabalhadores administrativos, totalizando 20 voluntários.

**Resultados:**

A prevalência de transtorno mental comum entre os profissionais da saúde e demais trabalhadores da saúde foi de 36%. Houve associação entre os domínios do Health Safety Executive – Indicator Tool e a prevalência de sofrimento psíquico nos profissionais de saúde nas variáveis apoio dos colegas, apoio da chefia e comunicação. No grupo de trabalhadores da saúde, essa associação também foi observada nas variáveis demanda, relacionamento, controle, apoio da chefia e comunicação.

**Conclusões:**

Os dados qualitativos apontaram a relevância dos fatores psicossociais associados ao sofrimento psíquico. Espera-se que a devolutiva destes resultados para a instituição de saúde desencadeie ações concretas da organização/gestão do trabalho em implementar estratégias para minimizar os estressores psicossociais que afetam a saúde e o bem-estar dos trabalhadores.

## INTRODUCTION

The International Labour Organization (ILO) and the World Health Organization (WHO)^1^ define psychosocial work factors (PWF) as the result of interactions between collective aspects — such as the work environment and organizational conditions — and individual worker characteristics, including skills, needs, culture, and personal circumstances. These factors can influence health, performance, and job satisfaction.^2^

The WHO considers PWF to include human resource management, alignment with organizational goals, communication and feedback methods, autonomy, social support, workload and work pace, environment and equipment, organizational culture, workplace interpersonal relationships, the role of the organization, career development, and work-home interface.^3^

Psychosocial risks, in turn, refer to the negative aspects of PWF that, if poorly managed, can lead to mental health issues among workers. These risks encompass specific conditions that increase the likelihood of stress, psychological distress, and other harmful consequences. Examples of psychosocial risks include excessive workload, lack of control over tasks, job insecurity, insufficient social support, workplace interpersonal conflicts, and constant pressure.

Certain occupational groups are more exposed to psychosocial risks, with health care workers — especially those in hospital setings — being particularly vulnerable. The impact of the COVID-19 pandemic^4^ further intensified their exposure to long working hours, excessive workloads, and hazardous conditions, including risks of accidents and exposure to infectious disease.^5^ Additionally, their daily tasks require direct contact with patients’ pain and illness, which can lead to emotional overload.^6^

Nurses, nursing technicians, and nursing assistants are recognized as among the most vulnerable to work-related psychological distress due to factors such as unclear professional roles, excessive workload resulting from staff shortages, and limited autonomy in decision-making, etc. Such elements can lead to chronic stress and burnout syndrome.^7,8^

A study conducted by Carvalho et al.^9^ on nursing staff highlighted the persistent workload demands in this profession. Among the participants, 152 (72%) reported biological work demands, 119 (56.4%) identified psychological demands, 117 (55.5%) noted physiological demands, 112 (53.1%) mentioned chemical demands, 105 (49.8%) experienced physical demands, and 75 (35.5%) faced mechanical demands.

In this study, we hypothesize hospital workers experience both intense workload pressures — stemming from excessive work hours, high-paced tasks, staff shortages, and performance targets — and a lack of recognition and autonomy as well as workplace conflicts, all of which contribute to psychological strain. Such factors may negatively impact workers and trigger mental health disorders.

The objectives of this study are to investigate the psychosocial work factors affecting professionals and workers in a general hospital in the interior of the Brazilian state of São Paulo and to assess the prevalence of psychological distress and associated factors. Findings aim to support the development of mental health promotion and prevention strategies.

## METHODS

This is a cross-sectional, quantitative-qualitative study based on the principles of action research, in which both the researcher and participants actively engage with the research problem,^10^ considering fieldwork as a process of discovery and creation.^11^ The study participants were workers from a general hospital located in the interior of the state of São Paulo, all of whom were invited to participate voluntarily. A total of 310 hospital employees were invited, including health care professionals engaged in direct patient care as well as support and administrative staff. The final sample consisted of 157 participants, ensuring approximately 90% sample reliability.

Among the participants, 87 were health care professionals, including nursing staff (nurses, technicians, and assistants) and other professionals such as psychologists, nutritionists, and physical therapists. A total of 67 were health care workers, including receptionists, administrative assistants, cleaning staff, laundry workers, and other hospital administrative personnel.

For data collection, a sociodemographic questionnaire,^8^ the Health Safety Executive – Indicator Tool (HSE-IT),^12^ and the Self-Reporting Questionnaire (SRQ-20) were applied. Data collection began after participants read and agreed to take part in the study, providing their informed consent by signing an informed consent form.

The sociodemographic questionnaire, adapted from the biopsychosocial questionnaire by Moreira & Lucca,^8^ consisted of 27 questions covering sex, marital status, age, race/color, disabilities, dependents, education level, workplace and department, job role, length of employment at the institution and in the current role, work schedule, shift, overtime work, occurrence of health issues (accidents and occupational diseases), experiences of moral and/or sexual harassment, use of psychotropic drugs, alcohol, and other substances, as well as aspects of job satisfaction regarding the workplace, the institution, leadership, and colleagues.

The SRQ-20 is a scale recommended by the WHO for assessing common mental disorders (CMDs). Developed by Harding et al.^13^ and validated in Brazil by Mari & Williams,^14^ it consists of 20 questions covering physical and psychological symptoms, with dichotomous response options (yes/no). Although it does not provide a psychiatric diagnosis, the SRQ-20 is useful for screening and generating diagnostic hypotheses. A cutoff score of 7 was adopted for analysis.^15^

The HSE-IT assesses psychosocial work factors, focusing on aspects of work design and management that may contribute to psychological or physical harm to workers.^11^ This questionnaire consists of 35 questions distributed across seven dimensions: demands (workload and job requirements), control (degree of autonomy), communication about changes, managers’ support, peer support, workplace interpersonal relationships, and job role. For each question, participants select one response from five options: (0) never, (1) seldom, (2) sometimes, (3) often, and (4) always, based on their perception of psychosocial work factors over the past 6 months.

In the results, work stress factors were determined by summing the scores of responses within each psychosocial work dimension. A score higher than 2 in the demands and relationships dimensions, and a score lower than 2 in control, managers’ support, peer support, job role, and communication dimensions, indicated psychosocial risk and occupational stress.

Volunteer participants were then invited to take part in focus groups (FGs), aiming to listen to workers and identify additional stressors in the workplace through a qualitative approach. According to Lucca & Sobral,^12^ the formation of FGs allows for a deeper listening to workers’ narratives, a beter understanding of real work processes, and the identification of relevant aspects not captured in the quantitative assessment.

A question guide was developed to structure FGs interviews, which were conducted in the hospital auditorium. Three FGs were held. The first one took place on June 21, 2024, with nursing coordinators, involving six participants. The second one was conducted on June 25, 2024, with health care workers, including eight participants: two receptionists, two cleaning staff, two kitchen assistants, one hospital laundry worker, and one laundry assistant. The third one was held on June 26, 2024, with six nursing technicians.

Data collection began after approval from the Human Research Ethics Commitee (opinion 6.339.713) under CAAE no. 74050823.5.0000.5496.

## RESULTS

Of the 157 respondents, 127 were women and 30 were men. Regarding age distribution, 18 participants were between 18 and 24 years old, 55 were between 25 and 34 years old, 71 were between 35 and 54 years old, and 13 were 55 or older. Concerning employment ties, 35 participants (22%) reported having two jobs.

The quantitative analysis of the HSE-IT instrument, based on the average data of the sample (Chart 1), did not identify any psychosocial dimensions as major stressors in the workplace, except for lack of control among health care professionals.

**Chart 1:** Average scores for psychosocial dimensions of HSE-IT related to work stress, Adamantina, 2024

HSE-IT psychosocial dimensions	Stress indicator	Total average (µ) of participants	Average (µ) of health care workers	Average (µ) of health care professionals
Demands	> 2	1.22	1.36	1.11
Control	< 2	2.01	2 .11	1.95
Managers’ support	< 2	2.67	2.46	2.84
Peer support	< 2	2.92	2.84	2.82
Relationships	> 2	1.35	1.36	1.35
Job role	< 2	3.32	3.23	3.41
Communication	< 2	2.50	2.31	2.64

HSE-IT = Health Safety Executive – Indicator Tool.

Among health care workers, control (*µ* = 2.11), communication (*µ* = 2.31), and managers’ support (*µ* = 2.46) have averages remarkably close to the cutoff score. Among health care professionals, only Control (*µ* = 1.95) is identified as a work stress factor.

In the percentage distribution of results, 47.13% of participants identified lack of control over decisions as a work stress factor, followed by communication issues (28.66%), lack of managers’ support (19.10%), interpersonal conflicts (14.01%), lack of peer support (11.46%), work demands (10.82%), and job dissatisfaction (2.54%). The descriptive analysis of the SRQ-20 questionnaire revealed 56 participants scored ≥ 7, indicating a 35.66% prevalence of psychological distress. Additionally, nine individuals reported having thoughts of ending their own life.

Sociodemographic factors are known to influence the occurrence of psychological distress. However, in this study, no significant relationship was found between sex, age group, length of employment at the institution, or having multiple jobs and the presence of psychological distress, either in the group of health care professionals or in the group of health care workers.

On the other hand, a significant association was found between the psychosocial dimensions of the HSE-IT and the SRQ-20 among health care professionals (Table 1), particularly in relation to lack of peer support, lack of managers’ support, and communication issues.

**Table 1:** Association between HSE-IT domains and the prevalence of psychological distress (SRQ-20) among health care professionals, Adamantina, 2024

Variable	Group	n	Mean	SD	Min	Max	p-value
Demands	SRQ-20 < 7	57	1.01	0.59	0.00	2.50	0.050
	SRQ-20 ≥ 7	31	1.30	0.66	0.12	2.75	
Relationships	SRQ-20 < 7	57	1.25	0.77	0.00	3.00	0.075
	SRQ-20 ≥ 7	31	1.54	0.71	0.00	3.25	
Control	SRQ-20 < 7	57	2.08	0.87	0.00	3.83	0.103
	SRQ-20 ≥ 7	31	1.73	0.95	0.33	3.83	
Managers’ support	SRQ-20 < 7	57	3.17	0.72	1.20	4.00	< 0.001
	SRQ-20 ≥ 7	31	2.27	0.84	0.40	3.60	
Peer support	SRQ-20 < 7	57	3.26	0.64	1.50	4.00	< 0.001
	SRQ-20 ≥ 7	31	2.53	0.91	0.50	4.00	
Job role	SRQ-20 < 7	57	3.50	0.44	2.40	4.00	0.058
	SRQ-20 ≥ 7	31	3.26	0.61	1.60	4.00	
Communication	SRQ-20 < 7	57	2.90	1.03	0.00	4.00	0.002
	SRQ-20 ≥ 7	31	2 .19	0.94	0.33	4.00	

SD = standard deviation; HSE-IT = Health Safety Executive – Indicator Tool; Max = maximum; Min = minimum; n = number of observations; SRQ-20 = Self-Reporting Questionnaire.

A significant association was found between the psychosocial dimensions of the HSE-IT and the SRQ-20 among health care workers (Table 2) in the following variables: demands, relationships, control, managers’ support, and communication (p = 0.004).

**Table 2:** Association between HSE-IT domains and the prevalence of psychological distress (SRQ-20) among health care workers, Adamantina, 2024

Variable	Group	n	Mean	SD	Min	Max	p-value
Demands	SRQ-20 < 7	45	1.17	0.57	0.00	2.62	< 0.001
	SRQ-20 ≥ 7	24	1 .74	0.53	0.37	2.87	
Relationships	SRQ-20 < 7	45	1.22	0.68	0.00	2.75	0.040
	SRQ-20 ≥ 7	24	1.66	0.88	0.00	3.50	
Control	SRQ-20 < 7	45	2.33	0.89	0.33	3.83	< 0.001
	SRQ-20 ≥ 7	24	1.71	0.60	0.83	2.83	
Managers’ support	SRQ-20 < 7	45	2.78	0.75	1.00	4.00	< 0.001
	SRQ-20 ≥ 7	24	1.88	0.78	0.20	3.40	
Peer support	SRQ-20 < 7	45	2.89	0.83	0.75	4.00	0.576
	SRQ-20 ≥ 7	24	2.76	0.94	1.00	4.00	
Job role	SRQ-20 < 7	45	3.25	0.65	1.20	4.00	0.767
	SRQ-20 ≥ 7	24	3.20	0.65	1.80	4.00	
Communication	SRQ-20 < 7	45	2.58	0.88	0.00	4.00	0.004
	SRQ-20 ≥ 7	24	1.82	1.05	0.00	4.00	

SD = standard deviation; HSE-IT = Health Safety Executive – Indicator Tool; Max = maximum; Min = minimum; n = number of observations; SRQ-20 = Self-Reporting Questionnaire.

## DISCUSSION

The association between psychosocial work factors, assessed through the HSE-IT questionnaire and focus groups, and the prevalence of SRQ-20 scores provided insights into the aspects and dimensions that contribute to either intensifying or mitigating psychological distress among health care workers.

The prevalence of CMDs among Brazilians is high, ranging from 17% to 77%.^16^ In this study, the prevalence of CMDs — indicating psychological distress — among participants was 35.66%. Several psychosocial factors are associated with higher CMD rates, including high job demands combined with low control, high expectations with litle support from peers and managers,^17^ interpersonal conflicts, individualism, professional disputes,^18^ imbalanced job design, uncertainty, and a lack of respect and workplace values.^19^

With the regionalization of the studied hospital, there was an increase in the number of patients served, leading to greater demands on direct care services as well as on hygiene, organization, material replacement, linen washing, room preparation, and food services, all while facing a shortage of staff to meet these new demands. Additionally, health care professionals struggle with bureaucratic tasks, such as filling out medical records and reports. The lack of adequate equipment, such as computers, further exacerbates the workload and delays administrative tasks.

Identification with patient care emerges as a central aspect of such professionals’ work. A nursing technician expressed this sentiment by stating, “*We don’t choose nursing; nursing chooses us*” (S18). When asked about the meaning of their work, health care professionals responded, “*Overall, saving lives*” (S15), “*Caring...*” (S16), and “*Love for others*” (S17). Caring for others provides a sense of gratification and symbolic reward, even in the face of hardship. As one nursing technician reflected, “[…] *in the end, we do it more out of love, right?*” (S18).

Therefore, although health care professionals identify job demands as a major psychosocial stressor, their connection to the nature of their work — specifically, caring for patients’ health and having some control over that care — helps regulate their mental well-being. These aspects are linked to the job role and control dimensions.

On the other hand, we found an association between psychosocial dimensions such as work relationships, lack of supervisor and colleague support, poor communication, and the perception of psychological distress. According to work psychodynamics theory,^20^ supportive relationships, both hierarchical and among peers, serve as an important symbolic and subjective factor in preventing psychological suffering and illness. Recognition, more than just material compensation, represents a symbolic reward that individuals expect and value in their work environment.

Health care professionals identified the lack of hierarchical recognition as a factor contributing to subjective demotivation. They also frequently mentioned team conflicts arising from a lack of cooperation, which leads to work overload due to the uneven distribution of tasks. One nursing technician described this frustration: “*That’s what I mean — sometimes the real suffering is that you’re the only one overwhelmed while others just stay in your shadow*” (S17, nursing technician).

Individuals reported that although they make suggestions to both direct and higher management to improve work organization such proposals are not considered. The lack of managers’ support contributes to team conflicts, fostering feelings of insecurity and injustice in the workplace. This environment also encourages peer rivalry and competition, further undermining morale and intensifying psychological distress at work.

Professionals reported hostility in communication between managers and subordinates, characterized by verbal atacks, embarrassment, and public exposure. Those favored by managers were also perceived to engage in equally hostile behavior toward their peers. Additionally, there was a sense that tasks were deliberately made more difficult, and in some cases, professionals were even prevented from performing their duties.

Situations of persecution, humiliation, and exclusion in the workplace were reported along with authoritarian relationships between managers and subordinates, which contribute to psychological distress at work. A nursing technician shared her experience of exclusion: “*[…] I have personally felt very excluded. […] Whatever they were going to do, whatever they were going to talk about, they did it among themselves, and I was left alone. Over time, we adapted, and things changed, but at first, that’s how it was*” (S16).

Health care workers, although they recognize the importance of their roles, do not feel acknowledged, often reporting experiences of invisibility in the workplace. One receptionist described this feeling: *“We at the reception desk deal with the tension from both patients and doctors, but our role is often not recognized”* (S11, receptionist). However, peer support serves as a protective factor. Many workers emphasized peer cooperation and solidarity in the workplace. A hospital laundry worker shared: “[…] *when I finish my tasks, I change clothes and go help the girls*” (S7, hospital laundry worker). A cleaning assistant echoed this sentiment: “[…] *after finishing our work, we always help each other*” (S13, cleaning assistant), while a food service worker described it as “[…] *like a chain, right?*” (S9, food service worker). Regarding job roles, health care workers have clearly defined tasks, which provides a greater sense of control and reduces pressure, as long as they fulfill their responsibilities.

Health care workers reported a significant increase in workload across their departments. In the laundry sector, the volume processed rose from 10,000 to 17,000 kilograms, while food distribution saw approximately a 50% increase. The high patient turnover and large number of hospital admissions have intensified the work pace, making it a major stressor. Additionally, the increased workflow has further impacted task control, as work has become even more affected by task unpredictability.

We found that workplace communication and relationships are often characterized by control and surveillance among peers, especially between workers from different departments. This dynamic, rooted in the culture of the organization, leads to interference in peer tasks and conflicts caused by distorted perceptions of reality. Communication failures between departments compromise task execution and, most importantly, the quality of patient care. This issue generates intense stress and ethical distress,^20^ as workers are often forced to conduct poorly managed tasks.

One example is the account of a food service worker who relies solely on memory to identify patients and distribute the correct diet: “[…] *if we don’t memorize the patient, we might give food to someone who isn’t allowed to eat it*” (S9, food service worker). This situation shifts the responsibility for the task onto the individual, rather than ensuring it through a safe organizational strategy, leading to stress, fatigue, and workplace distress.

Physical workload is also a significant stress factor, particularly in the laundry sector. One worker described the harsh conditions: “*The heat is unbearable; we spend 12 hours in a hot environment*” (S7). Additionally, the lack of adequate rest facilities during breaks hampers physical and mental recovery, further impacting workers throughout their shifts.

Workplace relationships with employees from other departments and managers are often conflict-ridden. One laundry assistant described the pervasive pressure: “*There isn’t a specific kind of pressure, but it feels like it’s coming from all sides, like a shadow*” (S8, laundry assistant). Health care workers also reported managers ignore their suggestions. A food service worker expressed frustration: “[…] *sometimes we have more insight, we want to speak up, but we aren’t heard, right?*” (S9, food service worker).

Regarding job roles, workers emphasized that well-defined tasks with slight variation help them gain mastery over their activities. A cleaning assistant explained: “[…] *a task is assigned to me, I have my role to perform, and if I do everything right, there’s no pressure*” (S13, cleaning assistant). Another food service worker added: “[…] *we follow the plan, we already know what to do and how to proceed*” (S9, food service worker).

The lack of association between psychological distress and colleague support highlights the protective role of peer relationships in the workplace. According to Dejours,^20^ recognition from coworkers can act as a barrier against psychological distress and mental illness, as it reinforces the individual’s role within the organization and strengthens social bonds in the workplace. This protective factor was frequently mentioned by workers who reported cooperative and supportive relationships with colleagues in their departments. A laundry worker shared: “*We spend the whole day laughing*” (S7, hospital laundry worker), while a food service worker added: “*Same here, we get along well*” (S10, food service worker).

One limitation of this study is that although the sample was significant within the context analyzed, the results may not be fully generalizable to other organizational setings.

## CONCLUSIONS

This mixed-method study, which included focus groups and the application of two questionnaires — the HSE-IT, assessing perceived psychosocial work factors (PWF) that trigger stress, and the SRQ-20, measuring psychological distress — investigated the working conditions of 157 health care professionals and workers in a medium-sized hospital.

The prevalence of CMDs was 35.66%, indicating that more than one-third of health care professionals and workers experience psychological distress. Among health care professionals, lack of colleague and supervisor support and poor communication were significantly associated with psychological distress. For health care workers, high job demands, strained work relationships, lack of control, and lack of managers’ support were the PWFs most strongly associated with psychological distress.

FGs narratives highlighted protective factors for mental health. Among health care professionals, identification with their work and the meaning they atribute to patient care played a crucial role in mobilizing aspects of subjectivity, providing symbolic rewards and identity gratification. For health care workers, clearly defined tasks and support networks from colleagues and immediate supervisors fostered cooperation and solidarity within the workforce.

We hope that sharing these findings with the health care organization will lead to concrete organizational and managerial actions to implement strategies aimed at reducing psychosocial stressors and improving the health and well-being of workers.
